# *Eristalis* flower flies can be mechanical vectors of the common trypanosome bee parasite, *Crithidia bombi*

**DOI:** 10.1038/s41598-021-95323-w

**Published:** 2021-08-04

**Authors:** Abby E. Davis, Kaitlin R. Deutsch, Alondra M. Torres, Mesly J. Mata Loya, Lauren V. Cody, Emma Harte, David Sossa, Paige A. Muñiz, Wee Hao Ng, Scott H. McArt

**Affiliations:** 1 Department of Entomology, Cornell University, Ithaca, NY 14853 USA; 2 Department of Environmental and Rural Science, The University of New England, Armidale, NSW 2351 Australia

**Keywords:** Community ecology, Entomology

## Abstract

Flowers can be transmission platforms for parasites that impact bee health, yet bees share floral resources with other pollinator taxa, such as flies, that may be hosts or non-host vectors (i.e., mechanical vectors) of parasites. Here, we assessed whether the fecal-orally transmitted gut parasite of bees, *Crithidia bombi*, can infect *Eristalis tenax* flower flies. We also investigated the potential for two confirmed solitary bee hosts of *C. bombi*, *Osmia lignaria* and *Megachile rotundata*, as well as two flower fly species, *Eristalis arbustorum* and *E. tenax,* to transmit the parasite at flowers. We found that *C. bombi* did not replicate (i.e., cause an active infection) in *E. tenax* flies. However, 93% of inoculated flies defecated live *C. bombi* in their first fecal event, and all contaminated fecal events contained *C. bombi* at concentrations sufficient to infect bumble bees. Flies and bees defecated inside the corolla (flower) more frequently than other plant locations, and flies defecated at volumes comparable to or greater than bees. Our results demonstrate that *Eristalis* flower flies are not hosts of *C. bombi*, but they may be mechanical vectors of this parasite at flowers. Thus, flower flies may amplify or dilute *C. bombi* in bee communities, though current theoretical work suggests that unless present in large populations, the effects of mechanical vectors will be smaller than hosts.

## Introduction

Recent analysis of long-term sampling data and biological records have shown that globally, wild insect pollinators, including solitary bees and flies, are experiencing population declines and range contractions^1–4^. Parasites are key drivers of pollinator health and are associated with declines of several pollinator species^5–7^. This fact is concerning for both conservation and economic reasons; pollination services are valued at more than $170 billion/year globally^8^, solitary bees and flies perform a large proportion of the services^9–12^, and agricultural dependence on pollinators continues to increase each year^13^.

Most information about pollinator parasites is known from social bees^7,14,15^. Honey bees (*Apis* spp.) and bumble bees (*Bombus* spp.) were previously thought to be the only host of certain viruses, microsporidians and trypanosomes, but recent studies have found many of these same parasites are present in and can infect wild solitary bee species, too^16–22^. The host range of these parasites, however, is understudied, and limited studies have assessed the incidence and infectivity of bee parasites in non-bee pollinators.

Evison et al.^23^ reported high prevalence of *Wolbachia* bacteria and *Ascosphera* fungi, and low prevalence of microsporidian fungi^23^, among wild-caught flower flies and bees. Additionally, Bailes et al.^24^ found high viral titres of two honey bee viruses (Sacbrood Virus and Black Queen Cell Virus) in wild-caught *Eristalis* (Diptera: Syrphidae) flower flies^24^. The nucleotide sequences of these viruses were 87–100% similar to those found in co-foraging honey bees, suggesting the viruses were not strains unique to flies and possibly being shared between the two taxa. Similarly, Brettell et al.^25^ also found bee-associated viruses in wild-caught flower flies after deep sequencing^25^. While these studies suggest many bee parasites may be broad, multi-host parasites, they do not show whether infection (active replication of the parasites) is occurring in non-bee pollinators, nor how transmission occurs.

The trypanosome gut parasite *Crithidia bombi* (Lipa & Triggiani 1988) lacks cell-specific host requirements compared to intracellular parasites, such as microsporidian *Nosema* spp*.*^26–28^, and has been found in a wide variety of solitary and social bees^15,21,22^. Despite historically being considered a “bumble bee parasite,” *C. bombi* was recently found to replicate in two species of solitary bees, the alfalfa leafcutter bee, *Megachile rotundata* (Fabricius 1787), and blue orchard bee, *Osmia lignaria* (Say 1837)^21,22^. The rather broad host requirements of *C. bombi* indicate this parasite may be able to infect, or pass through, the guts of many different flower-visiting insects. However, beyond the studies mentioned above, it is unknown if other flower-visiting insects such as flower flies (Diptera: Syrphidae) can act as hosts of *C. bombi*. This is important since flower flies occupy a similar ecological niche as bees because of their similar morphology, behaviors and foraging habits^29,30^.

In addition to host competence, epidemiology of multi-host parasites is also shaped by the environment. One particularly relevant factor to pollinator parasites are flowers—key hubs of transmission. Graystock et al.^31^ experimentally demonstrated that infected honey bees can transmit common, fecal–oral bee parasites including *C. bombi* to susceptible bumble bees (and vice versa) simply by foraging on the same flowers. Furthermore, a recent study that screened nearly 3000 flowers in nature found one in eleven flowers harbored pollinator parasites, including *C. bombi*^15^. Multiple factors such as landscape simplification^32^, presence of managed social bees^18^, floral traits^33,34^, and location of parasites on flowers^35^ can influence the prevalence and likelihood of transmission of pollinator parasites at flowers. Flies may contribute to transmission by mechanically spreading parasites from contaminated flowers, potentially redistributing the parasites on flowers during defecation and therefore creating more floral transmission hotspots. However, whether flies can act as mechanical vectors and transmit pollinator parasites at flowers, at quantities that can infect bees, is unknown. In addition, how flies compare to bees as parasite transmitters at flowers is also unknown.

As adult *Eristalis* flies visit the same floral resources as bees, they, too, can encounter, ingest and potentially become infected by common fecal-orally transmitted “bee” parasites. Therefore, we assessed: (1) whether the common, fecal-orally transmitted “bee” parasite, *C. bombi*, could infect the cosmopolitan European drone fly, *Eristalis tenax* (Linneaus 1758), (2) whether the quantity of viable *C. bombi* cells defecated by *E. tenax* would be sufficient to infect a common host of *C. bombi*, the common eastern bumble bee *Bombus impatiens* (Cresson 1863), and (3) whether two species of *Eristalis* flies, *Eristalis arbustorum* (Linneaus 1758) and *E. tenax,* as well as two megachilid bee hosts, *M. rotundata* and *O. lignara*, defecate on flowers and therefore could potentially transmit *C. bombi* at flowers.

## Results

### Evaluating whether the European drone fly, *Eristalis tenax*, is a host or non-host vector of *Crithidia bombi*

#### Inoculation of *Eristalis tenax* with *Crithidia bombi*

**Figure 1:**
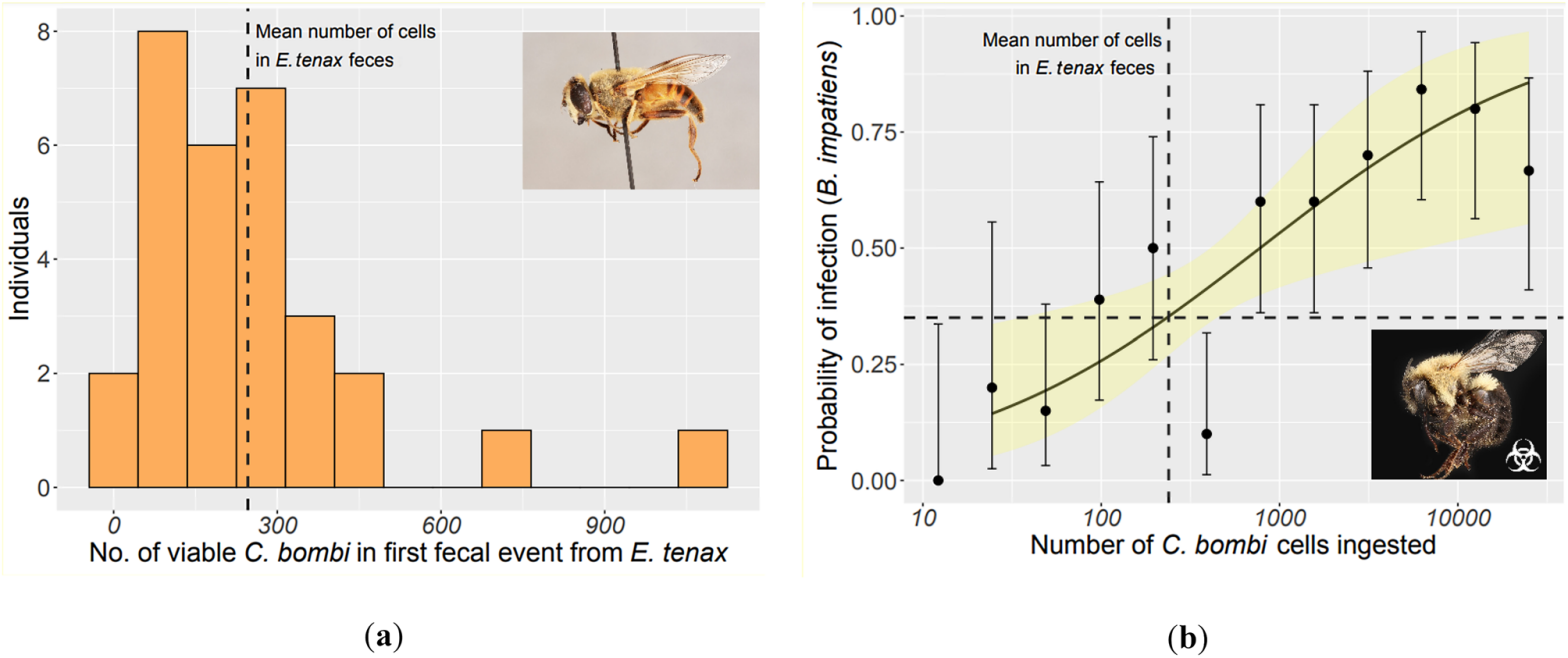
Flower flies can defecate enough live *Crithidia bombi* cells to infect bumble bee hosts: (**a**) Total number of *C. bombi* cells found in the first fecal events of inoculated *Eristalis tenax* flies (*n* = 30); (**b**) Probability of *C. bombi* infection of inoculated *Bombus impatiens* bumble bees (*n* = 9–12 per dose). In both figures, the vertical dotted lines indicate the mean number of *C. bombi* (239 cells) defecated in the first fecal events of *E. tenax* flies*.* In figure (**b**), the horizontal dotted line indicates that the probability of infection at 239 cells is 35%. 95% confidence intervals are shown as error bars for each treatment and the shaded area on the dose–response curve. “Side view of *Eristalis tenax*” image by Ken Perry, licensed under CC0 1.0. “Side view of *Bombus impatiens*” image by Christopher Johnson, licensed under CC0 1.0.

*Eristalis tenax* flies were inoculated with *C. bombi* and both the first defecation event and gut were screened for the parasite. *C. bombi* was never found in the gut of the flies 10 days post-inoculation (Table 1). However, all flies defecated within 5 hours of the start of the experiment and the first fecal event from 93% of the inoculated flies (*n* = 30) contained live *C. bombi* (Table 1). The average amount of live *C. bombi* in these first fecal events were 239 parasites (95% CI 174.4–362.7 parasites; Fig. 1a). One fecal event contained a total of 1080 cells, which was the greatest concentration and equated to roughly one-third of the inoculum fed to the flies. There was no significant difference in *C. bombi* load between males vs. females (LRT, χ^2^_1_ = 0.19, p = 0.66) and no *C. bombi* were found in the first defecation events of the control flies (*n* = 30; Table 1). The average first fecal volume among the inoculated flies was 1.21 μL (95% CI 1.03–1.39 μL; Supplementary Figure S1). Together, these results indicate that *C. bombi* cells can survive passage through fly digestive tracts, although they do not cause active infections in the flies.

**Table 1:** Proportion of inoculated and control *Eristalis tenax* flies with live *Crithidia bombi* cells in first fecal events (day 1) and guts 10 days post-inoculation.

Treatment	Live *C. bombi* in fly feces (day 1)	Live *C. bombi* in fly gut (day 10)
Control	0/30	0/30
Inoculated	28/30	0/30

#### Response of *Bombus impatiens* to varying doses of *Crithidia bombi*

Bumble bees (*B. impatiens* workers) from two colonies were inoculated with varying doses (between 12 to 25,000 cells) of *C. bombi* as shown in Fig. 1b. These doses were chosen to include the realistic range of *C. bombi* in flower fly feces (shown above) and beyond. Infection probability increased with dose, and the slope of the relationship was colony-dependent (dose-colony interaction: LR = 9.6, *p* = 0.002, Supplementary Figure S2a). Smaller bees were also more likely to become infected (LR = 14, *p* < 0.001). Among the infected bees, infection intensity increased with dose (*t*_69_ = 2.8, *p* = 0.007), was also colony-dependent (*t*_69_ = − 2.122, *p* = 0.037, Figure S2b), and smaller bees had higher infection intensities (*t*_69_ = − 2.8, *p* = 0.006). Conditional (pseudo-)*R*^2^ of the models were 0.43 for infection probability, and 0.27 for infection intensity.

We generated dose–response curves for the two responses by marginalizing across colony and bee size (see Fig. 1b for infection probability, and Supplementary Figure S3 for infection intensity). Marginal (pseudo-)*R*^2^, based on dose alone, was 0.27 for infection probability and 0.15 for infection intensity. Generalized linear models assume linear relationships between the link function and predictor. For each response and each colony, we did not find substantially stronger support for more flexible monotonic additive models (infection probability: ΔAIC = − 3 × 10^–4^ (colony 1), 1.2 (colony 2); infection intensity: ΔAIC = 0.016 (colony 1), 1.3 (colony 2), suggesting that the linear relationships were adequate at capturing the shape of the dose–response curves.

We found that all treatments but the 12-cell dosage of *C. bombi* could infect bumble bees. The lowest infective dose (24 cells) had a 14% likelihood of bee infection and the highest dose (25,000 cells) had an 85% likelihood of bee infection. When we mapped the levels of *C. bombi* found in inoculated flower fly feces to the likelihood of infection using the dose–response curve, we found that all flower fly feces containing viable *C. bombi* could potentially infect *B. impatiens*. The lowest quantity of *C. bombi* parasites defecated by a fly was 60 cells, which had a 24% likelihood of bee infection (Fig. 1b). The highest quantity of *C. bombi* parasites defecated was 1080 cells, corresponding to a 54% likelihood of infection if consumed by *B. impatiens*. The mean quantity of *C. bombi* parasites (239 cells) corresponds to a 35% likelihood of infection if consumed by *B. impatiens* (Fig. 1b).

### Vectoring potential of two bee species, *Osmia lignaria* and *Megachile rotundata*, and two *Eristalis* fly species, *E. arbustorum* and *E. tenax*

#### Fecal volumes and defecation frequency

**Figure 2:**
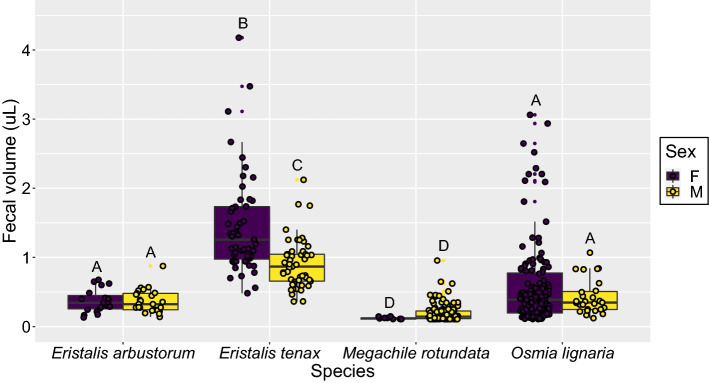
Fecal volumes of two flower fly pollinators, *Eristalis arbustorum* and *E. tenax*, and two bee pollinators, *Megachile rotundata* and *Osmia lignaria*. Whiskers indicate the range of fecal volumes, excluding outliers. Upper, middle and lower quartiles indicate the greatest, average and lowest fecal volumes collected, respectively. Data points have been jittered for clarity. Different letters indicate significant pairwise post-hoc contrasts (*p* < 0.05).

All pollinators were placed in individual cages lined with filter paper and fed ad libitum sucrose solution with fluorescent powder to measure fecal volumes and defecation frequency in a 24-h period. When comparing fecal volumes between different species and sex using the filter paper data only (which used identical methodology for all pollinators), we found that the species-sex interaction was significant (*F*_(3, 457)_ = 10, *p* < 0.001; Fig. 2), so main effects were not tested in accordance to principle of marginality. Based on post-hoc pairwise contrasts (Supplementary Table S1), we found that for both sexes, *E. tenax* had significantly larger fecal volumes than the three other pollinator species, while *E. arbustorum* and *O. lignaria* both had larger fecal volumes than *M. rotundata* (Fig. 2). Within each species, only *E. tenax* showed a significant difference between sexes, with females having larger fecal volumes than males.

For *E. tenax* only, comparing the fecal volumes from the two methods (collected from microcentrifuge tubes in the inoculation experiment vs. estimated from filter paper spot diameters), we found that the method-sex interaction was marginally significant (*F*_(1,126)_ = 3.9, *p* = 0.052), while both the main effects of method and sex were significant (method: *F*_(1,127)_ = 6.0, *p* = 0.016; sex: *F*_(1,127)_ = 22, *p* < 0.001). In particular, we found that fecal volumes collected from the microcentrifuge tubes were about 25% (95% CI 4.6–50%) larger than fecal volumes estimated using standard curves from filter paper spot diameters (Supplementary Figure S4). Nonetheless, since the interaction was not significant, this means that any inference about fecal volume differences between groups should not be affected by the choice of methods.

**Figure 3:**
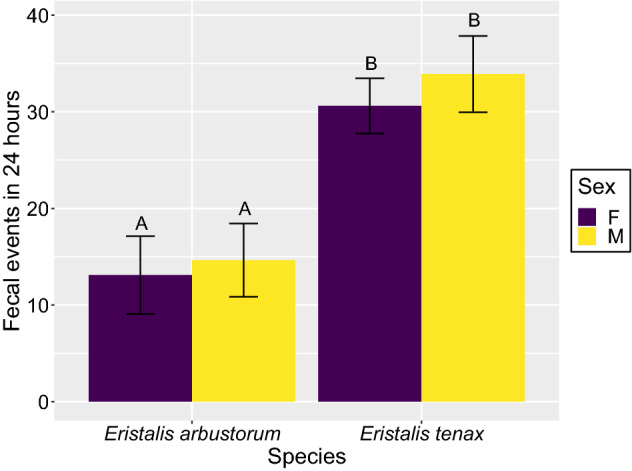
Number of fecal events by *Eristalis arbustorum* and *E. tenax* flies over 24 h. Different letters indicate post-hoc significance (*p* < 0.05), error bars indicate 95% confidence intervals.

*Eristalis tenax* flies defecated more frequently than *E. arbustorum* flies in a 24-h period (*F*_(1, 137)_ = 85, *p* < 0.001; Fig. 3). Uninfected *E. arbustorum* flies defecated, on average, 14 times in a 24-hour period and uninfected *E. tenax* flies defecated, on average, 32 times in a 24-hour period. Neither the species-sex interaction nor sex were important predictors of defecation frequency of *E. arbustorum* and *E. tenax* flies (*F*_(1, 136)_ = 0.24, *p* = 0.63; *F*_(1, 137)_ = 2.2, *p* = 0.14).

#### Defecation patterns on a shared floral resource

**Figure 4:**
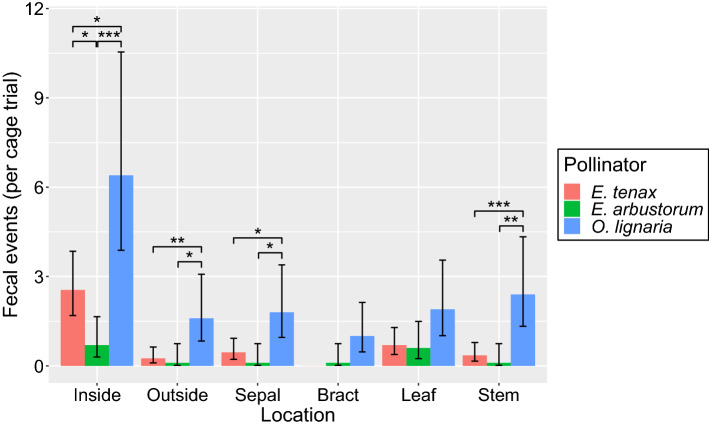
Number of *Eristalis tenax*, *Eristalis arbustorum* and *Osmia lignaria* fecal events per cage trial (*n* = 10 each) at six different floral locations (inside the corolla, outside the corolla, on the sepal, bract, leaf or stem) of *Solidago dansolitum* ‘Little Lemon’ goldenrod. Asterisks indicate Tukey-corrected significance of pairwise contrasts between species within each location (***p < 0.001; **p < 0.01; *p < 0.05). Error bars indicate 95% confidence intervals.

Defecation events were recorded on *Solidago dansolitum* ‘Little Lemon’ goldenrod in all cage trials with *E. tenax* flies (*n* = 20 trials) and *O. lignaria* bees (*n* = 10 trials), while *E. arbustorum* defecated on *Solidago* in only 8 of 10 trials. Both *E. arbustorum* and *O. lignaria* defecated on all locations of *Solidago*, while *E. tenax* defecated on all locations but the bract (Fig. 4). The interaction between pollinator species and plant location was not significant (LRT, χ^2^_10_ = 16, *p* = 0.093), while both species and location main effects were significant (LRT, χ^2^_2_ = 88, *p* < 0.001; χ^2^_5_ = 90, *p* < 0.001). Post-hoc tests indicate that *E. tenax* defecated on *Solidago* more often than *E. arbustorum* (*Z*-test, *Z*_(Inf)_ = 2.778, *p* = 0.015), and *O. lignaria* defecated on *Solidago* more often than *E. tenax* and *E. arbustorum*, respectively (post-hoc *Z*-test, *Z*_(Inf)_ = − 7.261, *p* < 0.001; *Z*_(Inf)_ = − 7.446, *p* =  < 0.001).

We found that the inside of the corolla was defecated on most often (compared to the outside of the corolla, sepal, bract, leaf and stem) respectively (*p* < 0.001 in each case; Fig. 4). In addition, the leaf was defecated on more frequently than the bract (post-hoc *Z*-test, *Z*_(Inf)_ = − 3.542, *p* = 0.005).

## Discussion

In this study, we found that *C. bombi* did not replicate and cause an active infection in *E. tenax* flies. However, 93% of inoculated flies defecated live *C. bombi* in their first fecal event, often at levels capable of infecting bumble bees. In addition, we show that *Eristalis arbustorum* and *E. tenax* both defecate comparable or larger volumes, respectively, of feces compared to *Megachile rotundata* and *Osmia lignaria*, two solitary bees that are recently confirmed hosts of *C. bombi*^21,22^. Furthermore, *E. tenax* and *E. arbustorum* are both shown to defecate on flowers, which are indirect transmission routes for *C. bombi*^31,35,36^. Taken together, these results indicate that while *Eristalis* flower flies are not hosts of *C. bombi*, they can potentially be non-host vectors (i.e., mechanical vectors) that contribute to community-wide transmission of this multi-host parasite.

Infected bumble bees are known to shed similar parasite loads to the inoculum we fed to *E. tenax* flies^37–39^. In fact, heavily infected bumble bees can shed concentrations as high as 55,000 cells/μL in their feces^39^, suggesting flies can encounter and potentially ingest much higher *C. bombi* loads than the 3200 cells we used for inoculum. We found that inoculated *E. tenax* flies defecated *C. bombi* in levels lower than those ingested. However, all *C. bombi* quantities found in the fly feces were capable of establishing an infection in bumble bees. Specifically, we show that an inoculation dosage of only 24 *C. bombi* cells can establish an infection in bumble bees. This quantity of *C. bombi* is less than the lowest quantity we found in flower fly feces (60 cells) and much lower than the mean *C. bombi* quantity in flower fly feces (239 cells). As susceptible hosts range in size and immune-related traits, the number of parasites required to infect a smaller host, such as *M. rotundata or O. lignaria,* may be different compared to larger *Bombus* hosts. Therefore, the infectivity of *C. bombi* loads shown in this study may vary based on the susceptibility of host species.

While we report that *C. bombi* does not infect *E. tenax* flies, uninfected *Eristalis* flies still possess traits which increase their vectoring potential of the parasite by defecating frequently and in large volumes. *E. arbustorum* flies defecated comparable volumes to *O. lignaria* bees*,* and *E. tenax* flies defecated the largest fecal volumes of the four pollinators. Although both bees are competent hosts and therefore vectors of *C. bombi*^21^, our results suggest that *O. lignaria* may be more likely to transmit the parasite, as this bee species defecates larger fecal volumes than *M. rotundata.* Similarly, our results suggest that of the two fly species tested, *E. tenax* may be more likely to vector the parasite because this species defecated larger fecal volumes and more frequent fecal events than *E. arbustorum.* However, this may be attributed to *E. tenax* being observationally larger in size than *E. arbustorum*. Susceptible pollinators are more likely to acquire fecal-orally transmitted parasites from large volumes of infected feces when foraging, as large fecal events take longer to evaporate, thus allowing parasites to survive for a greater period of time outside of a host^35^. When these infected fecal events are also defecated frequently, susceptible hosts have an even greater chance of encountering these parasites. Differences in vectoring potential between competent hosts and non-host vectors warrant further investigation, with the possibility to reveal novel factors that may be incorporated into disease modelling of species-rich pollinator communities^40^.

We also demonstrated that bee and fly pollinators defecate on certain locations of a shared floral resource more frequently than others. The bee *O. lignaria* defecated the most on goldenrod*,* which may suggest this known host of *C. bombi* spent the most amount of time on the floral resource compared to the two fly species. However, of the six locations (inside the corolla, outside the corolla, on the sepal, bract, stem and leaves), all three pollinators defecated most often on the inside of the corolla. Goldenrod is an important late-season resource for many pollinators, and since *Crithidia* is not a vertically transmitted parasite^41^, solitary pollinators foraging on flowers for pollen and nectar are likely acquiring the parasite at shared floral resources.

While *E. tenax* flies can act as mechanical vectors by ingesting and shedding viable *C. bombi* cells, this does not necessarily imply an amplification in disease transmission. When vectors ingest parasites at a floral resource, they also remove them from existing floral hotspots; mechanical vectors redistribute parasites across the landscape by increasing the number of hotspots, but at the expense of decreasing the average parasite load per hotspot. Whether this redistribution leads to amplification from the increased number of hotspots, or a dilution from the decreased average load, depends on the dose–response relationship between host and parasite and the number of non-host vectors capable of spreading the parasite^42^. The successful infection of bumble bees when fed low inoculum doses (e.g., only 24 cells) of *C. bombi*, however, suggest the potential for amplification. The unsuccessful infection of *Eristalis* flies, coupled with the flies shedding fewer parasites than ingested (e.g. less than one-third the 3200 cell inoculum) in first fecal events, suggest the potential for dilution of the parasite. Although ultimately both these hypotheses will need to be assessed using a quantitative epidemiological model.

Furthermore, it is unknown how many non-host vectors can spread bee parasites at flowers. Ruiz-Gonzalez & Brown^43^ showed that honey bees are possible non-host vectors of *C. bombi,* as they demonstrated the parasite can survive passing through the guts of honey bees (as demonstrated here with *Eristalis* flower flies), and can still be infective to bumble bees. Also, *C. bombi* has been found on the external surface of uninfected bumble bees sampled in natural field conditions^44^. Here we suggest including flies, when appropriate, in pollinator disease transmission dynamics, as the roles of flower-visiting flies has often been speculated but not tested. Cook et al.^45^ found that flies from 86 families have been reported visiting the flowers of more than 1100 different species of plants globally, however, flies are not the only insects sharing floral resources with bees.

When we assessed *C. bombi* viability in the feces of the *E. tenax* flies, we deemed the parasites viable if they were still motile. While the parasites were all actively swimming, we anecdotally observed that the parasites found in fly feces swam with less vigor compared to those that were harvested from bumble bee guts. Whether this impacts likelihood of infecting new hosts is unknown and beyond the scope of our current study. However, we suggest assessing the infectivity of *Eristalis-*defecated *C. bombi* for known hosts, such as *Bombus* spp., *O. lignaria* and *M. rotundata* bees. More broadly, little is known regarding the infectivity of mechanically transmitted fecal–oral parasites that pass through non-host vectors.

Although flower flies may vector bee parasites, the ecological services these flies provide as adults (pollination) and as larvae (predation and decomposition) still make them essential non-bee pollinators to support in ecosystems^45,46^. Doyle et al.^47^ found that flower flies visit at least 72% of global agricultural food crops estimated to be worth US$300 billion annually, making flies the second most important pollinator taxon behind bees. Fly pollinators, however, are historically understudied compared to bees. We suggest investigating the ecological services they may provide, as well as comparing the efficacy of flies and bees in different crop systems to make an informed decision on their role in pollinator disease transmission dynamics.

Our findings provide justification to look beyond bees to better understand the epidemiology of species-rich pollinator communities. We show that while *E. tenax* flies are not hosts of *C. bombi*, they can defecate viable *C. bombi* cells. However, *Eristalis* flies still possess traits that facilitate the dispersal of fecal-orally transmitted “bee” parasites, by defecating frequently and in large volumes inside the corolla of flowers where susceptible hosts forage for nectar and pollen which has important implications not only for *Crithidia* parasite transmission networks, but general plant-pollinator-parasite networks. Also, our results suggest that the vectoring potential of known hosts of *C. bombi* may vary between species, as *M. rotundata* defecated significantly smaller fecal volumes than *O. lignaria* bees. Therefore, we recommend investigating whether more non-bee pollinators that share floral resources with bees can be hosts or non-host vectors of bee parasites so these species can be incorporated in epidemiological models of pollinator communities.

## Methods

### Rearing methodology

*Eristalis* flower flies were reared in laboratory conditions from egg clutches laid by wild-caught females in the summer of 2019 (see Supplementary Materials for detailed rearing methodology). Only flies that emerged on the same day were used in the experiments. An artificial diapause protocol (see Supplementary Materials for detailed protocol) was used to prolong the lifespan of lab-reared flower flies, as adult *Eristalis* flower flies in lab colonies have shorter lifespans than adult *Eristalis* flies in the wild^48^. Once the adult flies emerged, all siblings were placed in artificial diapause in a refrigerator and fed 10% sucrose ad libitum until the experiment began. These *Eristalis* flower fly rearing and artificial diapause protocols are a modification of previously published protocols^48,49^.

*Osmia lignaria* (*n* = 50; Crown Bees, Woodinville, WA, USA) and *Megachile rotundata* (*n* = 50; Watts Solitary Bees, Bothell, WA, USA) were purchased and allowed to emerge in an incubator kept at 23 °C and 65% humidity*.* Bumble bees (*Bombus impatiens*) used as *C. bombi* source colonies or as uninfected sources of bees for the dose–response trials were purchased from Biobest (Biobest, Leamington, Ontario, Canada) and maintained in the lab by feeding sucrose and pollen from a mixture of honey bee-collected poly-floral pollen (Bee Pollen Granules, CC Pollen High Desert, Phoenix AZ, USA). To ensure the commercial colonies were free of parasites, we pulled 20 workers and screened them for parasites via microscopy. No parasites were found in any of the colonies used for the dose–response trials.

### Evaluating whether the European drone fly, *Eristalis tenax*, is a host of *Crithidia bombi*

After breaking artificial diapause, the *E. tenax* flower flies were allowed to groom, but not feed, for one hour. Each fly was then placed abdomen-first into a 1.5 mL microcentrifuge tube harness to collect defecation events (Supplementary Figure S5). The size of these tubes allowed the flies to feed comfortably, but the tubes were also tapered at the bottom, which prevented the flies from stepping in their feces. Holes were placed along the side of the tubes so the fly could respire. One large hole was placed on the lid of the tube so the fly could be inoculated directly with a pipette.

Flies were randomly divided into treatment and control groups. *E. tenax* flies in a roughly 1:2 F:M sex ratio were used in both the treatment (*n* = 30) and control groups (*n* = 30), for a total of 60 replicates. The flies that emerged from the same egg clutch with this 1:2 F:M sex ratio were the only siblings that could accommodate the replicates needed for this experiment, which is why this sex ratio was used.

The *C. bombi* inoculum was made fresh from infected *B. impatens* individuals the morning of the experiment using established protocols. Briefly, we dissected the gut of infected *B. impatiens* workers from a laboratory source colony that sustained a strain collected from wild *B. impatiens* workers from Massachusetts, USA (GPS coordinates: 42.363911 N, – 72.567747 W). We homogenized the bee guts in distilled water and diluted the mixture to 1280 *C. bombi* cells μL^−1^, which we then combined 1:1 with 30% sucrose solution for an inoculum of 640 cells μL^−1^, a standard inoculum concentration for infecting bumble bees with *C. bombi*^35,50^. Control groups were fed 5 μL of a 30% sucrose and blue dye (Butler Extract Co., Lancaster, PA, USA) that in pilot experiments was not found to influence host or parasite survival. Treatment groups were inoculated with 5 μL (3200 cells total) of *C. bombi*, 30% sucrose and blue dye solution. The number of cells used in the inoculum is similar to levels of *C. bombi* found in the feces of bumblebees with recently established infections^37^. Blue dye was used to better visualize when fecal events occurred and flies that did not drink the entire 5 μL inoculum were not used in the experiment.

After feeding, the flies were monitored continuously until defecation occurred. As these flies recently emerged from artificial diapause and were starved pre-experiment, every hour post-inoculation the flies were fed a 30% sucrose and blue dye solution ad libitum to encourage defecation. Once a fly defecated, the feces were collected via pipette and diluted to a 10 μL solution with deionized water to observe and count parasites using Kova Glasstic slides. The fly was then placed in an individual 60 mL plastic portion cup with filter paper (Sigma–Aldrich, St Louis, MO, USA) and a 1.5 mL microcentrifuge tube feeder containing 500 μL of a 30% sucrose and blue dye solution for 10 days. Feeders and filter papers were replaced every 3 days to prevent mold growth. As *C. bombi* typically replicates in high numbers after 10 days in the guts of bumble bees^51^, both control and treatment flies were dissected and *C. bombi* gut counts were performed 10 days post-inoculation. Since actively swimming, and thus live, *C. bombi* is infective to susceptible bumble bee hosts^35^, only actively swimming *C. bombi* were counted. The fecal volume, dilution factor and counts of *C. bombi* were quantified for each individual fly to calculate the exact amount of *C. bombi* in the individual’s first defecation event.

### Dose–response data

*Crithidia bombi* inoculum was made from infected *B. impatiens* individuals the morning of each trial using the protocols described above, with two exceptions. First, the *C. bombi* strain was collected from wild *B. impatiens* workers from New York, USA (GPS coordinates: 42.457350, − 76.426907). Second, a range of serially diluted doses were used to inoculate uninfected *B. impatiens* workers. The doses were: 25,000 cells, 12,500 cells, 6250 cells, 3125 cells, 1563 cells, 781 cells, 391 cells, 195 cells, 98 cells, 49 cells, 24 cells, and 12 cells. To obtain these doses, we homogenized bee guts in distilled water and diluted the mixture to 5000 *C. bombi* cells μL^−1^ with 30% sucrose solution. Serial dilutions were then conducted with a 10% sucrose solution to ensure the same osmolarity of each inoculum.

We conducted four replicate dose–response trials over a period of four weeks. Each week, five uninfected workers per dose from each of two colonies were administered 5 μL of *C. bombi* inoculum. The ten highest doses were administered for the first 2 weeks, and two additional doses (24 cells, and 12 cells) were added for the final 2 weeks. Inoculated bees were kept individually in vials and fed 30% sucrose ad libitum for 7 days at 23 °C and 65% humidity. After 7 days, the bees were dissected and *C. bombi* loads were quantified using a hemocytometer as described above. In addition, the right forewing was removed from each bee and marginal cell length was measured as a proxy for size^52^. In total, 220 bees were inoculated (20 replicates for each of the ten highest doses, 10 replicates for the two lowest doses).

#### Defecation patterns on a shared floral resource

All pollinators (*O. lignaria, M. rotundata, E. arbustorum* and *E. tenax*) were placed in individual 60 mL plastic portion cups lined with filter paper. Each pollinator received a 1.5 mL microcentrifuge tube feeder containing 500 μL of fluorescent dye via 2.5 g of fluorescent powder (Stardust Micas) dissolved into 500 mL 30% sucrose feeders to visualize fecal deposition on flowers. After 24 hours, filter papers were collected (for analysis of fecal volume and defecation frequency, see below) and a total of five, randomly selected pollinators of the same species were placed in 12 × 12 × 12″ mesh cages (Bioquip Products, Rancho Dominguez, CA, USA) containing inflorescences of similar sized *Solidago dansolitum* ‘Little Lemon’ goldenrod each replicate trial. Goldenrod was used in this experiment because both bees and flower flies were observed foraging on this abundant floral resource. Only pollinators with filter papers containing fluorescing defecation events were released in the mesh cages.

All *E. arbustorum* cages (*n* = 10) contained 2:3 F:M sex ratios, except one cage contained a 3:2 F:M sex ratio. All *E. tenax* cages (*n* = 20) contained 3:2 F:M sex ratios, except four cages contained 2:3 F:M sex ratios. All *O. lignaria* cages (*n* = 10) contained 4:1 F:M sex ratios, except one cage contained a 3:2 F:M sex ratio. For the two fly species, sample sizes and F:M sex ratios were determined by the greatest, same-day sibling emergence. For *O. lignaria,* sample sizes and F:M sex ratios were determined by emergence availability. *M. rotundata* floral deposition data was not collected, as the F:M emergence was heavily skewed to males that did not interact with, and therefore defecate on, the flowers.

After 24-hours, the pollinators were removed and the defecation events on the goldenrod from all cages were counted under a blacklight. The location of the defecation events on the goldenrod was recorded. The plant parts were divided into six categories: ‘inside’ the flower (inside the corolla), ‘outside’ the flower (surface of the corolla), on the sepal, on the bract (the leaflike structure beneath the flower), on the stem or on a leaf.

#### Defecation frequency and fecal volumes

The diameter of the smallest and largest defecation events per filter paper was measured by a digital caliper and an average diameter was calculated from these two values for all pollinators. The average diameter of the defecation events was converted to an average volume (in μL) using a standard curve (Supplementary Figure S6; *R*^2^ = 0.99 for the calibration data). The collected fecal volumes defecated by control flies from the *E. tenax* inoculation experiment (see above) were compared to the average fly fecal volumes calculated here. This was done to analyze whether flies in a confined environment, where they were inoculated with *C. bombi,* defecated similar volumes to flies allowed to move freely in an individual cup, which the average volumes were estimated from. In addition*,* the number of defecation events (frequency) over a 24-hour period on the collected *E. arbustorum* (*n* = 46) and *E. tenax* (*n* = 100) filter papers were counted for each fly.

### Statistical analyses

For the *E. tenax* inoculation experiment, we evaluated the amount of *C. bombi* cells in the first defecation event using a negative binomial generalized linear model (GLM), with fly sex as predictor. We chose negative binomial over Poisson to account for overdispersion, which we evaluated using Pearson residuals. Significance of sex was evaluated using a likelihood ratio test (LRT).

Data from the *B. impatiens* inoculation experiment were used to fit two dose–response curves, the first for infection probability, and the second for infection intensity among infected bees. Infection intensity was defined using the loads estimated from the hemocytometer. A bee was considered infected if the counts were nonzero. We first tested whether the dose ingested, wing length (as a proxy for body size) and the colony the bee came from affected its response. For infection probability, this was done using a GLM with log_10_(dose), colony, wing length and their interactions as predictors, and infection status as the Bernoulli response. For infection intensity, this was done using a linear model (LM) with the same predictors, and log_10_(intensity) as response, using only infected bees. Doses were log-transformed in accordance to how the experimental doses were varied, while intensities were log-transformed to achieve normality of the residuals. Significance of predictors were tested in accordance with the principle of marginality.

While we found that wing length and colony were significant predictors, in practice the colony-specific response of a wild bee is unknown (since it would not have come from any of the experimental colonies), while the dependence on wing length is only useful in a size-based epidemiological model. Hence, we generated dose–response curves by marginalizing across colony and wing length. Finally, we tested whether linear relationships between the link function and log_10_(dose), assumed in LMs and GLMs, were sufficient to capture the shape of the dose–response curves, by fitting the data to shape-constrained additive models and then comparing AIC values^53^. SCAMs are generalized additive models (GAMs) on which additional constraints such as monotonicity have been imposed; being more flexible, they can better capture the shapes of the dose–response curves should the linear relationships be inadequate.

We evaluated whether fecal volume depended on pollinator species and sex with a linear model (LM), fitted using weighted least squares to account for unequal variances between group (detected using Levene's test). Since the transformation from diameter (of feces on filter paper) to volume introduced a noticeable skew to the distribution, we transformed the volume back to diameter and further performed a Box-Cox transformation to achieve normality^54^, which we verified using the Shapiro–Wilk and D'Agostino's K^2^ test. The transformed volume was used as the response in the abovementioned linear model.

For *E. tenax*, fecal volume was also manually collected from the 1.5 microcentrifuge tubes during the inoculations experiment. We compared the fecal volume from the two methods using a LM with method and fly sex as well as their interaction as predictors. Volumes were log-transformed to achieve normality, while the linear model was fitted using ordinary least squares since Levene's test indicated no significant deviation from the assumption of equal variance.

We evaluated whether defecation frequency depended on pollinator species and sex with a LM, again fitted using weighted least squares to account for unequal variances between groups. While two of the groups showed deviation from normality using the Shapiro–Wilk test, the deviations were only marginally significant and hence not expected to qualitatively affect the results^55^.

Finally, we evaluated defecation patterns on goldenrod using a negative binomial GLM, with feces counts as the response, and pollinator species, plant location and their interaction as predictors. We did not use a mixed model with cage number as a random effect since there was only one count value per cage per location, so pseudo-replication was not an issue. Significance of predictors were evaluated using LRT in accordance to the principle of marginality^56^ (i.e., main effects were tested only when their interactions were insignificant and hence dropped). Post-hoc tests of pairwise contrasts with Tukey corrections were performed for predictors that were significant. We recognize that the principle sex ratio and its interactions with other predictors could also be included among the predictors; however, since each species had cages with predominantly one sex ratio (*E. tenax* 3F:2M; *E. arbustorum* 2F:3M; *O. lignaria* mix of 4F:1M and 5F:0M), this meant that species and sex ratios were highly correlated, making it impossible to separate their effects. Nonetheless, since female *Eristalis* flies do not provision their brood, the differences between sexes (e.g., time spent foraging on plants) may be less pronounced than in bees.

## Supplementary Information

Supplementary Information.
