# Supplementary material for: Eristalis flower flies can be mechanical vectors of the common trypanosome bee parasite, Crithidia bombi
Source: Sci Rep. 2021 Aug 4;11:15852. doi: 10.1038/s41598-021-95323-w (PMC8338921; doi:10.1038/s41598-021-95323-w)
Supplement: Supplementary file 1 — Supplementary Information. [file 41598_2021_95323_MOESM1_ESM.docx]

***Eristalis* flower flies can be mechanical vectors of the common trypanosome bee parasite, *Crithidia bombi***

Authors Abby E. Davis ^a, b^ * [aed236@cornell.edu](mailto:aed236@cornell.edu)
Kaitlin R. Deutsch ^a^ [krd59@cornell.edu](mailto:krd59@cornell.edu)
Alondra M. Torres ^a^ [alondra.torres.gonzalez@gmail.com](mailto:alondra.torres.gonzalez@gmail.com)
Mesly J. Mata Loya ^a^ [Mesly.J.Mata.Loya-1@ou.edu](mailto:Mesly.J.Mata.Loya-1@ou.edu)
Lauren V. Cody ^a^ [lvc26@cornell.edu](mailto:lvc26@cornell.edu)
Emma Harte ^a^ [evh27@cornell.edu](mailto:evh27@cornell.edu)
David Sossa ^a^ [davidemiro@gmail.com](mailto:davidemiro@gmail.com)
Paige A. Muñiz ^a^ [pam287@cornell.edu](mailto:pam287@cornell.edu)
Wee Hao Ng ^a^ [wn68@cornell.edu](mailto:wn68@cornell.edu)
Scott H. McArt ^a^ [shm33@cornell.edu](mailto:shm33@cornell.edu)

^a^ Department of Entomology, Cornell University, Ithaca, New York 14853, USA

^b^ Department of Environmental and Rural Science, The University of New England, Armidale, New South Wales 2351, AU

**Supplementary Information**

**Methodology**

**Rearing methodology for *Eristalis* flies**

To encourage female, wild-caught *Eristalis* flies to oviposit, the flies were placed in artificial diapause (see artificial diapause protocol below). Once in artificial diapause for four days, the flies were removed and placed in their own individual 60 mL plastic portion cup with filter paper (Sigma–Aldrich, St Louis, MO, USA) and a 1.5 mL microcentrifuge tube feeder containing 500 uL of 30% sucrose. This 60 mL plastic portion cup, 30% sucrose feeder and filter paper housing system will further be referred to in this paper as an individual cup. If the females were already mated, the artificial diapause typically encouraged oviposition on the filter paper within an hour after refrigerator removal. If the flies did not oviposit, they were released.


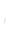
Egg clutches were collected with a fine-tipped paintbrush and placed in 1.5 mL microcentrifuge tubes. The microcentrifuge tube was then placed in a petri dish. To increase humidity, 30 mL of warm water was added to the petri dish. The egg clutches were not placed directly in the water. The petri dishes containing the eggs were sealed with Parafilm (Bemis Company Inc., Neenah, WI, USA) to encourage humidity and retain moisture. The *Eristalis* eggs hatched within 2-3 days when the petri dishes were kept in room temperature. To prevent desiccation, egg clutches were collected within one day of being laid.

The contents of the petri dish were placed in a 5 L bucket containing 4 L composted sheep manure (Shelterbelt Farms, Brooktondale, NY, USA) and 1 L tap water. This 4 L composted sheep manure and 1 L tap water mixture will further be referred to as the 5 L compost slurry. A nutritional supplement of activated yeast and water was added to the slurry when moisture levels looked visually depleted. The activated yeast nutritional supplement decreased competition for food between the larvae, therefore, the original 4 L composted sheep manure was never replaced for the entirely of the experiment. Once placed in the 5 L compost slurry, the *Eristalis* larvae pupated within 15 – 20 days.

The bucket of 5 L compost slurry was placed within a larger cardboard box. Dry, 100% organic compost (Arbico Organics, Tucson, AZ, USA) was added to the cardboard box, six inches from the rim of the 5 L bucket. Wood shavings were placed up to the rim of the bucket, which allowed third instar larvae to crawl out of the 5 L bucket and pupate in a dry location. The cardboard box containing the 5 L manure bucket was then placed inside a 24” x 24” x 36” mesh cage (Bioquip Products, Rancho Dominguez, CA, USA). The flies eclosed within 7-13 days of pupating when in room temperature (20 degrees Celsius). All lab-reared adult flies were collected daily and placed in artificial diapause.

This protocol successfully reared both *Eristalis tenax* and *E. arbustorum* flies.

**Artificial diapause protocol**

Adult *Eristalis* flies were placed in half-inflated gallon Ziploc bags in groups of 25 or less. Approximately 1.0 g pollen patty (30% sucrose and Bee-Pro pollen substitute (Mann Lake Ltd., Hackensack MN, USA)) and a three-inch cotton wick saturated with 30% sucrose was placed in a 60 mL plastic portion cup (HD Supply Facilities Maintenance, Burlington, NJ 08016) with the flies. If the cotton wick is too saturated, moisture will accumulate in the plastic bag, encouraging mold growth and negatively impacting fly survival. Pollen and sucrose were replenished as needed. The Ziploc bag was then placed in a refrigerator, at a temperature of 8-10 degrees Celsius, in darkness. The metabolism of the fly is suspended in this period of artificial diapause, causing significantly reduced feeding and grooming behavior.

Artificial diapause was broken every four days by removing the flies from the refrigerator. The flies were placed in a 13.5 x 13.5 x 24" mesh cage (Bioquip Products, Rancho Dominguez, CA, USA) for four hours to encourage grooming, flying and feeding. Flies that emerged (see rearing methodology for *Eristalis* flies below) on the same day were placed in the same mesh cage. These flies were then placed into a new gallon Ziploc bag and provided with fresh pollen and sucrose. The Ziplock bags were then placed back in a refrigerator. This cycle of repeatedly moving the flies in and out of the refrigerator every four days was repeated until the flies were used in an experiment.

**Tables**

Table S1: Post-hoc pairwise contrasts between fecal volumes of pollinator species, marginalized across sex.

| **Pairwise contrast** | Estimate | *t*_457_ | *p* value |
| --- | --- | --- | --- |
| *E. arbustorum* - *E. tenax* | -0.55 | -14 | < 0.001 |
| *E. arbustorum* - *M. rotundata* | 0.55 | 12 | < 0.001 |
| *E. arbustorum* - *O. lignaria* | -0.072 | -1.5 | 0.40 |
| *E. tenax* - *M. rotundata* | 1.1 | 30 | < 0.001 |
| *E. tenax* - *O. lignaria* | 0.47 | 13 | < 0.001 |
| *M. rotundata* - *O. lignaria* | -0.62 | -14 | < 0.001 |

**Figures**

**Figure S1**: Fecal volumes (in uL) of the first defecation event of *Eristalis tenax* flies (*n* = 30) that were inoculated with the bee-associated parasite, *Crithidia bombi.* The dotted line indicates the mean.

| 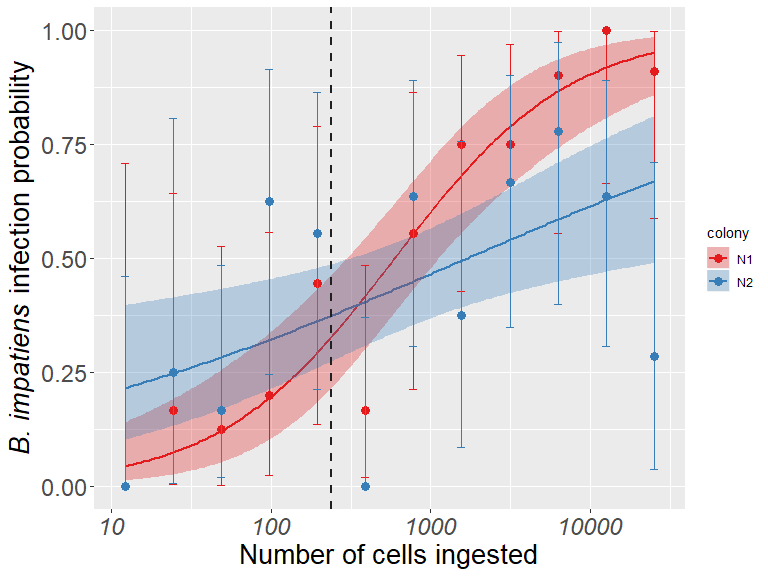  (**a**) | 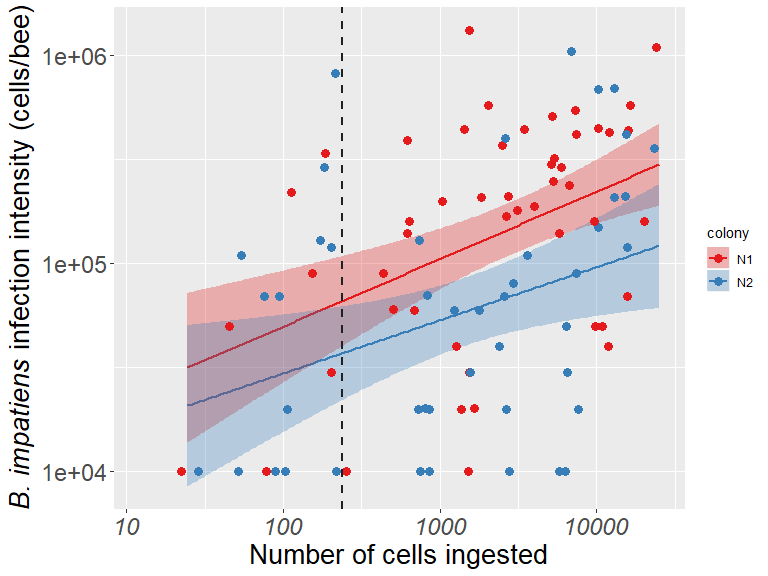  (**b**) |
| --- | --- |

**Figure S2:** Colony-specific dose-response curves: **(a)** infection probability and **(b)** infection intensity of infected *Bombus impatiens* bumble bees. Each color represents one colony. In (**a**), error bars are 95% Clopper-Pearson confidence intervals. Points in (**b**) have been jittered for clarity. In both panels, the shaded regions correspond to 95% confidence intervals of the predicted mean values. The dotted lines indicated the average number of *C. bombi* (239 cells) defecated in the first fecal events.


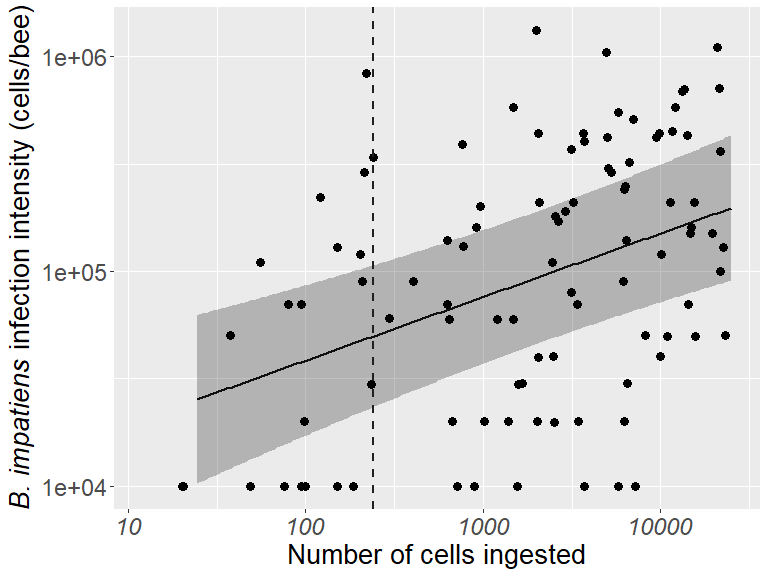


**Figure S3:** Dose-response relationship for infection intensity, marginalized across colonies. The shaded region corresponds to the 95% confidence intervals of the predicted mean values. Points have been jittered for clarity. The dotted lines indicated the average number of *C. bombi* (239 cells) defecated in the first fecal events.


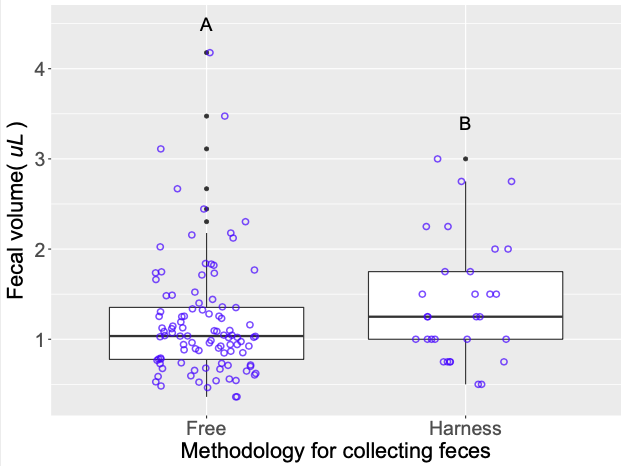


**Figure S4**: Comparing methodologies for collecting fly feces. “Free” indicated the fecal events were collected from filter papers. “Harness” indicated the fecal events were pipetted while the flies were immobile in a 1.5 mL microcentrifuge tube harness. Whiskers indicate the range of fecal volumes, excluding outliers. Upper, middle and lower quartiles indicate the greatest, average and lowest fecal volumes collected, respectively. Data points are jittered over the figures for clarity. Different letters indicate significant pairwise contrasts (*p* < 0.05).


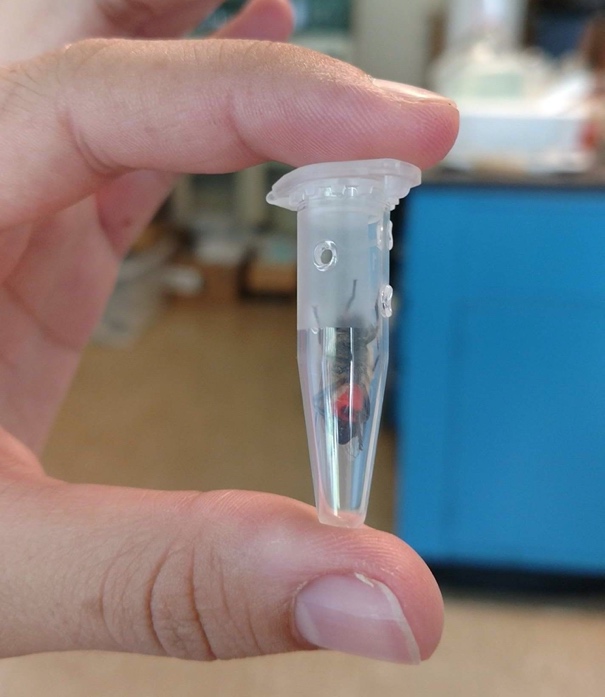


**Figure S5:** “Harness” system flies were placed in to collect defecation events. Flies were inoculated in these 1.5 microcentrifuge tubes by placing a hole on the lid of the tube and feeding the flies manually through the hole with a pipette. Red coloration of the abdomen is due to sucrose with dye having been consumed to ensure fecal events were more easily identifiable.


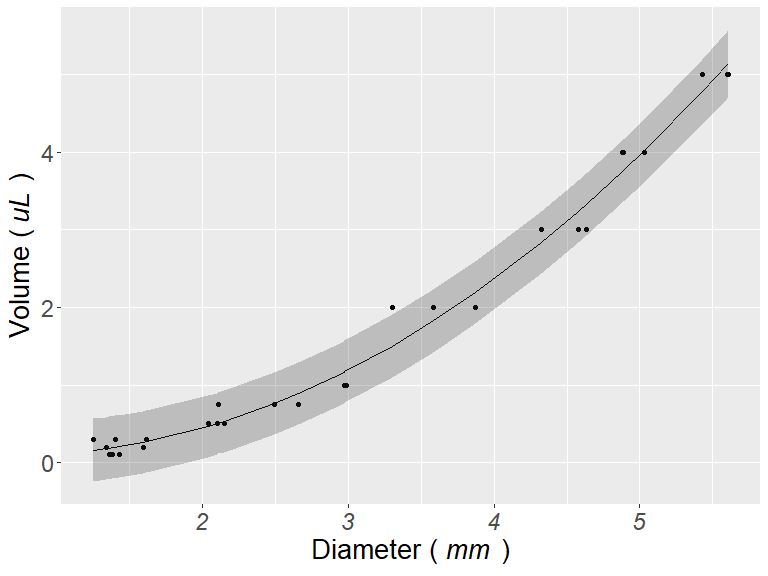


**Figure S6:** Standard curve of the average diameter (mm) of *Eristalis tenax* fly fecal events converted to average volume (in uL). The average of the smallest and largest fecal events (*n* = 28) were measured from filter papers. The shaded region corresponds to the 95% confidence intervals.
